# Epigenetic regulation of NfatC1 transcription and osteoclastogenesis by nicotinamide phosphoribosyl transferase in the pathogenesis of arthritis

**DOI:** 10.1038/s41420-018-0134-6

**Published:** 2019-02-06

**Authors:** Xuanan Li, Shamima Islam, Min Xiong, Ndona N. Nsumu, Mark W. Lee, Li Qin Zhang, Yasuyoshi Ueki, Daniel P. Heruth, Guanghua Lei, Shui Qing Ye

**Affiliations:** 1 Division of Experimental and Translational Genetics, Children’s Mercy, Kansas City, MO 64108 USA; 2 Department of Biomedical and Health Informatics, University of Missouri Kansas City School of Medicine, Kansas City, MO 64108 USA; 3 Department of Orthopedics, Xiangya Hospital, Central South University, Changsha, 410005 China; 4 Department of Chemistry, University of Missouri, Columbia, MO 65211 USA; 5 Department of Oral and Craniofacial Sciences, School of Dentistry, University of Missouri-Kansas City, Kansas City, MO 64108 USA

## Abstract

Nicotinamide phosphoribosyltransferase (NAMPT) functions in NAD synthesis, apoptosis, and inflammation. Dysregulation of NAMPT has been associated with several inflammatory diseases, including rheumatoid arthritis (RA). The purpose of this study was to investigate NAMPT’s role in arthritis using mouse and cellular models. Collagen-induced arthritis (CIA) in DBA/1J *Nampt*^+/−^ mice was evaluated by ELISA, micro-CT, and RNA-sequencing (RNA-seq). In vitro Nampt loss-of-function and gain-of-function studies on osteoclastogenesis were examined by TRAP staining, nascent RNA capture, luciferase reporter assays, and ChIP-PCR. Nampt-deficient mice presented with suppressed inflammatory bone destruction and disease progression in a CIA mouse model. Nampt expression was required for the epigenetic regulation of the Nfatc1 promoter and osteoclastogenesis. Finally, RNA-seq identified 690 differentially expressed genes in whole ankle joints which associated (*P* < 0.05) with Nampt expression and CIA. Selected target was validated by RT-PCR or functional characterization. We have provided evidence that NAMPT functions as a genetic risk factor and a potential therapeutic target to RA.

## Introduction

Rheumatoid arthritis (RA) is characterized by synovial inflammation and bone erosion^1,2^. Unfortunately, current therapies for arthritis are inadequate and there remains a need for additional therapeutic targets.

Nicotinamide phosphoribosyltransferase (NAMPT) is an essential gene^3^ which functions in NAD synthesis, apoptosis, and inflammation^4^. NAMPT is expressed in nearly all organs, tissues, and cells examined^4^. Because of its pleiotropic functions, dysregulated NAMPT expression has been implicated in the pathogenesis of several diseases, including arthritis, though the role of NAMPT in these disorders remains to be elucidated^4^. Using a collagen-induced arthritis (CIA) mouse model, Busso et al. demonstrated that FK866, a NAMPT inhibitor, effectively reduced the severity and progression of arthritis^5^. The progression of CIA was also slowed by selective siRNA knockdown of NAMPT in Ly6C^high^ monocytes^6^. However, no study has been conducted to systematically evaluate the molecular mechanisms of Nampt in arthritis in well-established Nampt knockdown (*Nampt*^+/−^) and Nampt overexpression (*Nampt*^OE^) mice to substantiate that Nampt is a genetic risk factor and potential therapeutic target in RA.

This study investigated the molecular mechanisms of Nampt in arthritis through integrative approaches of CIA mouse models, in vitro cellular experimentation, and transcriptional profiling. We validated Nampt’s involvement in arthritis using a CIA mouse model^7,8^ in wild-type and *Nampt* heterozygous knockdown DBA/1J mice, and we investigated further the pathways underlying Nampt’s mechanism in arthritis through loss-of-function and gain-of-function studies experiments in mouse bone marrow-derived macrophages (BMM) and RNA sequencing (RNA-seq) of CIA mouse tissue. Selected targets from RNA-seq discovery were experimentally validated. Our results support the hypothesis that NAMPT is a genetic risk factor and a potential therapeutic target in RA.

## Results

### Decreased inflammation and suppressed bone erosion in collagen-induced arthritis in DBA/1J *Nampt*^+/−^ mice

**Fig. 1:**
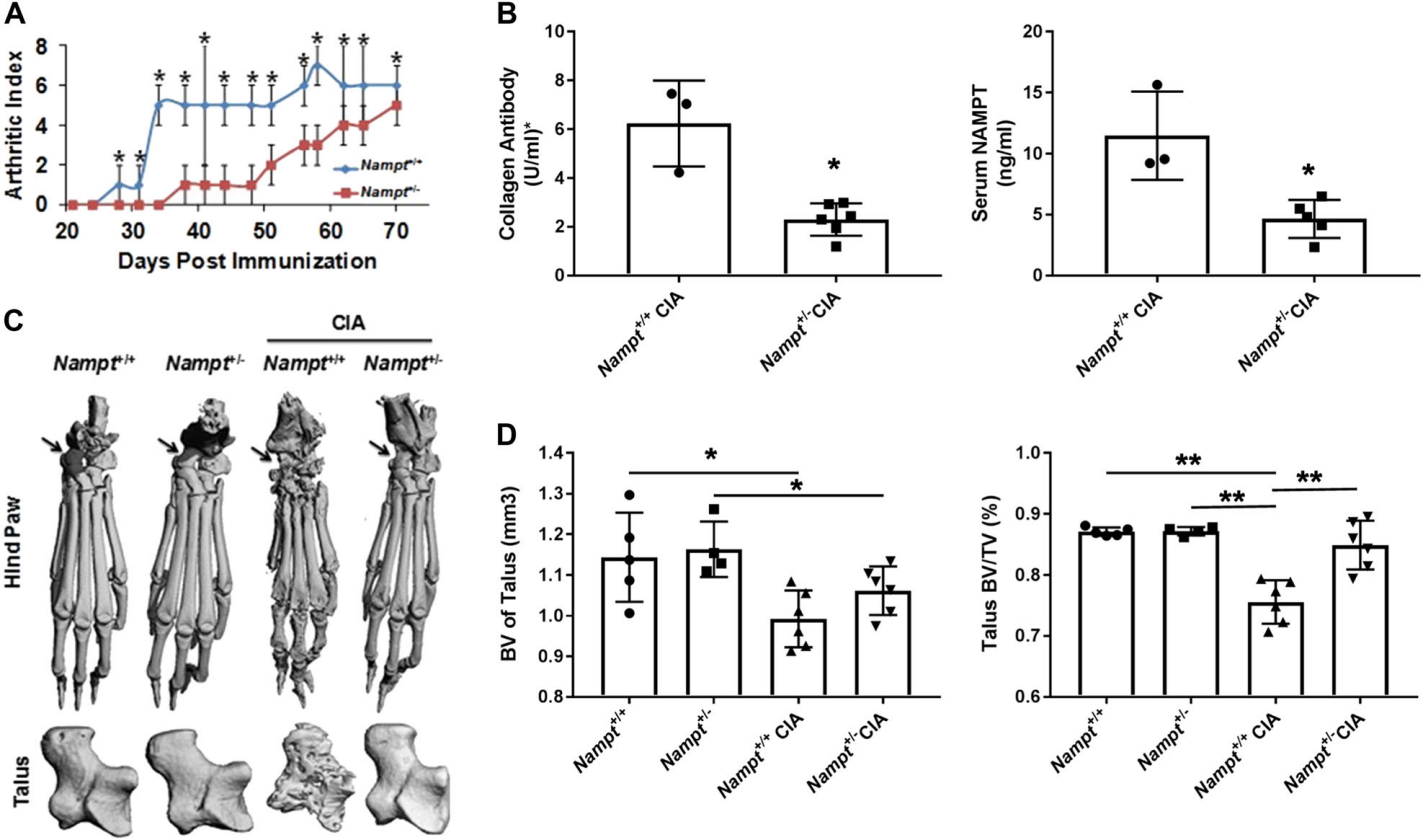
Decreased NAMPT expression attenuates inflammation and bone erosion in collagen-induced arthritis. CIA was induced in 8 weeks old, male DBA/1J *Nampt*^+/+^ and *Nampt*^+/−^ mice. Observable inflammation was observed for 10 weeks. Serum and hind limbs were collected for ELISA and micro-CT analysis, respectively. **a** Changes in joint inflammation scores in *Nampt*^+/+^ (*n* = 11) and *Nampt*^+/−^ (*n* = 13). Arthritic index score is reported as mean ± median absolute deviation. **b** Serum concentrations of anti-collagen antibody (scale ×100,000) and NAMPT. The results are presented as mean ± SD; *Nampt*^+/+^ CIA (*n* = 3), *Nampt*^+/−^ CIA (*n* = 5). **c** Representative micro-CT images of left hind paws and talus bones. Arrow represents the location of the talus in ankle joint. **d** Bone volume (BV) and bone volume per total volume (BV/TV) in talus are presented as mean ± SD; *Nampt*^+/+^ (*n* = 5), *Nampt*^+/−^ (*n* = 4), *Nampt*^+/+^ CIA (*n* = 6), 7 *Nampt*^+/−^ CIA (*n* = 6). *P < 0.05, **P < 0.005

To explore the molecular mechanisms of altered Nampt expression in arthritis, we characterized CIA in *Nampt*^+/−^ and *Nampt*^+/+^ mice. The progression of observable inflammation was less severe in *Nampt*^+/−^ mice compared to *Nampt*^+/+^ with a significant difference in the median arthritic index from day 28 to 70 post-immunization (Fig. 1a). The incidence of arthritis (index score > 1) was decreased slightly in *Nampt*^+/−^ mice (85%, 11/13) compared to *Nampt*^+/+^ (100%, 11/11). At day 70 post-immunization, the mice were euthanized for tissue isolation and sample analysis.

To elucidate potential mechanisms by which the induction of arthritis is suppressed in *Nampt*^+/−^ mice, we measured the serum levels of the arthritogenic anti-mouse CII auto-antibody, which plays a crucial role in the initiation of CIA^7^. The serum levels of the CII antibody were decreased significantly in *Nampt*^+/−^ mice compared with *Nampt*^+/+^ mice (Fig. 1b). The decreased immune response corresponded with lower levels of circulating Nampt in the heterozygous mice compared to wild-type mice (Fig. 1b).

To evaluate the effect of CIA on focal bone loss, we analyzed the hind paw and talus by micro-computed-tomographic (micro-CT) imaging. Micro-CT revealed visible differences between the hind paw and talus of *Nampt*^+/+^ and *Nampt*^+/−^ mice with CIA (Fig. 1c). To quantify bone loss, we contoured the talus to determine bone volume (BV). The average total BV of the talus were significantly lower in both CIA *Nampt*^+/+^ and *Nampt*^+/−^ mice compared with their non-immunized controls (Fig. 1d). Correction of the BV by the total volume of the talus (BV/TV) revealed bone loss was significantly milder in CIA *Nampt*^+/−^ compared with CIA *Nampt*^+/+^ mice (Fig. 1d).

### Attenuated osteoclastogenesis in Nampt-deficient primary BMM and RAW 264.7 cells

**Fig. 2:**
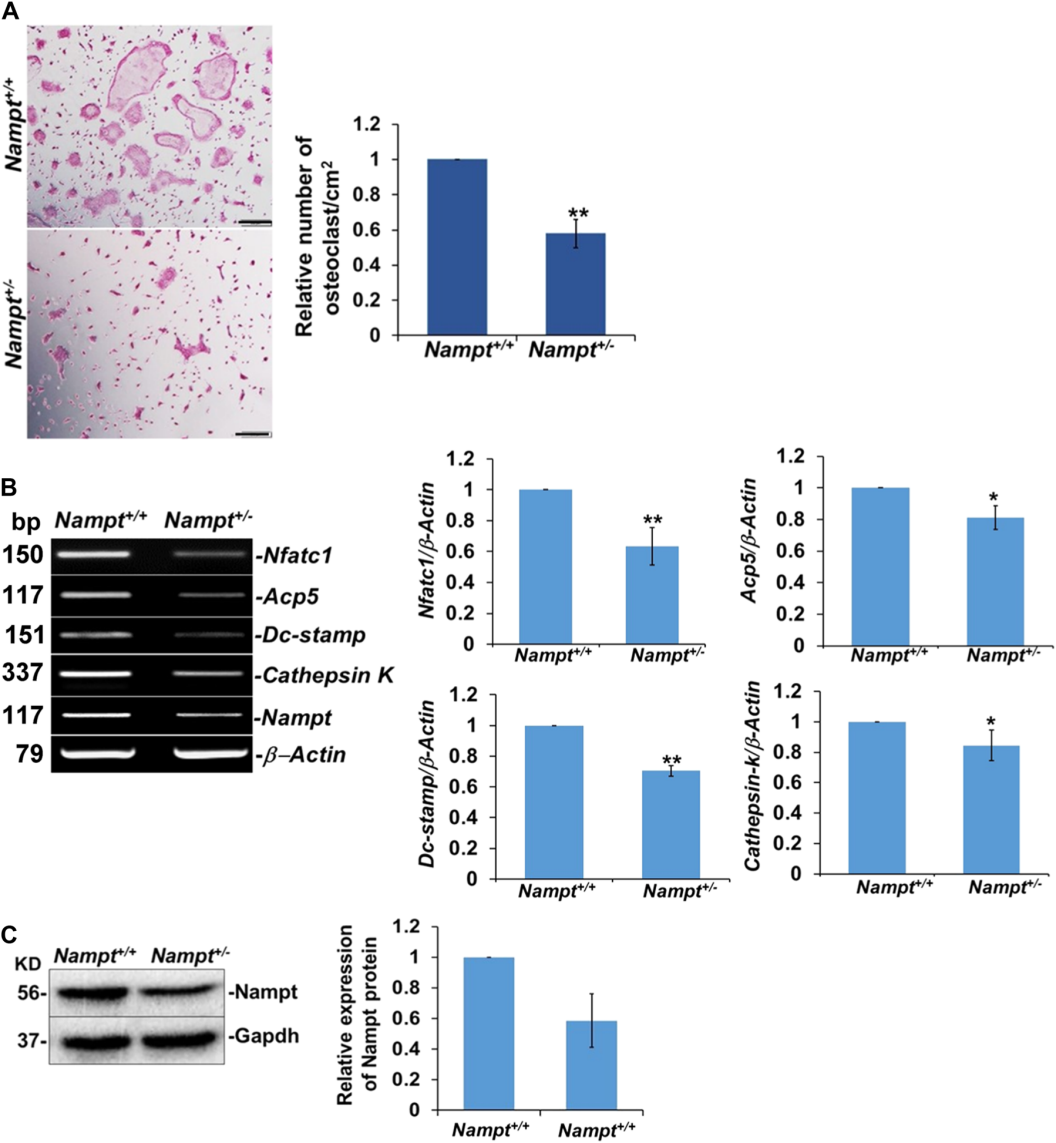
Inhibition of Rankl induced osteoclastogenesis in macrophages from *Nampt*^+/−^ mice. **a** Bone marrow-derived macrophages from *Nampt*^+/+^ and *Nampt*^+/−^ mice were treated with M-CSF (20 ng/ml) for 2 days. Non-adherent cells were washed out and cells were further cultured with M-CSF (20 ng/ml) and Rankl (100 ng/ml) for an additional 3 days. TRAP staining was performed 3 days later and cells having 3 or more nuclei were counted as osteoclasts. The frequency of osteoclasts is expressed as mean ± SD (*N* = 5). An average of 7 fields/well were counted per experimental sample. **b** Reduced osteoclast target gene expression in Nampt-deficient macrophages from *Nampt*^+/−^ mice. Osteoclast target *Nfatc1*, *Acp5*, *Dc-stamp*, *Cathepsin K* mRNA expression were measured using semi-quantitative RT-PCR. *β-Actin* was used as a loading control. **c** Representative western blot of Nampt protein in Nampt-deficient macrophages. Quantification of Nampt protein expression in Nampt-deficient macrophages. Representative images from three *Nampt*^+/−^ mice with *Nampt*^+/+^ littermate controls are presented. Quantification of semi-quantitative RT-PCR of osteoclast target genes expressed as mean ± SD (*N* = 3). *P < 0.05; **P < 0.005

Our finding that *Nampt*^+/−^ mice were protected against bone erosion in CIA led us to hypothesize that Nampt plays a critical role in osteoclast differentiation and that decreased Nampt expression attenuates osteoclast formation. To test this potential mechanism, we examined Rankl-dependent osteoclast differentiation in BMM isolated from *Nampt*^+/+^ and *Nampt*^+/−^ mice (Fig. 2). TRAP activity, a histochemical marker of osteoclastogenesis^9^, was detected by cell staining (Fig. 2a). M-CSF-dependent *Nampt*^+/+^ BMM were able to produce TRAP^+^ cells following a 3-day stimulation with M-CSF and Rankl, while stimulated *Nampt*^+/−^ BMM produced significantly fewer TRAP^+^ cells. The relative number of differentiated osteoclasts from *Nampt*^+/−^ BMM was significantly lower compared with *Nampt*^+/+^ BMM (Fig. 2a). Decreased differentiation of Nampt-deficient macrophages into TRAP^+^ cells correlated with lower expression of *Acp5*, the gene encoding TRAP protein. mRNA levels for key osteoclast markers, including *Nfatc*1, *Dc-stamp*, and *Cathepsin K*, were also lower in osteoclasts derived from *Nampt*^+/−^ BMM compared with *Nampt*^+/+^ controls, thus validating the attenuation of osteoclastogenesis (Fig. 2b). Western blot analyses verified that Nampt expression was decreased in *Nampt*^+/−^ BMM relative to *Nampt*^+/+^ BMM (Fig. 2c).

**Fig. 3:**
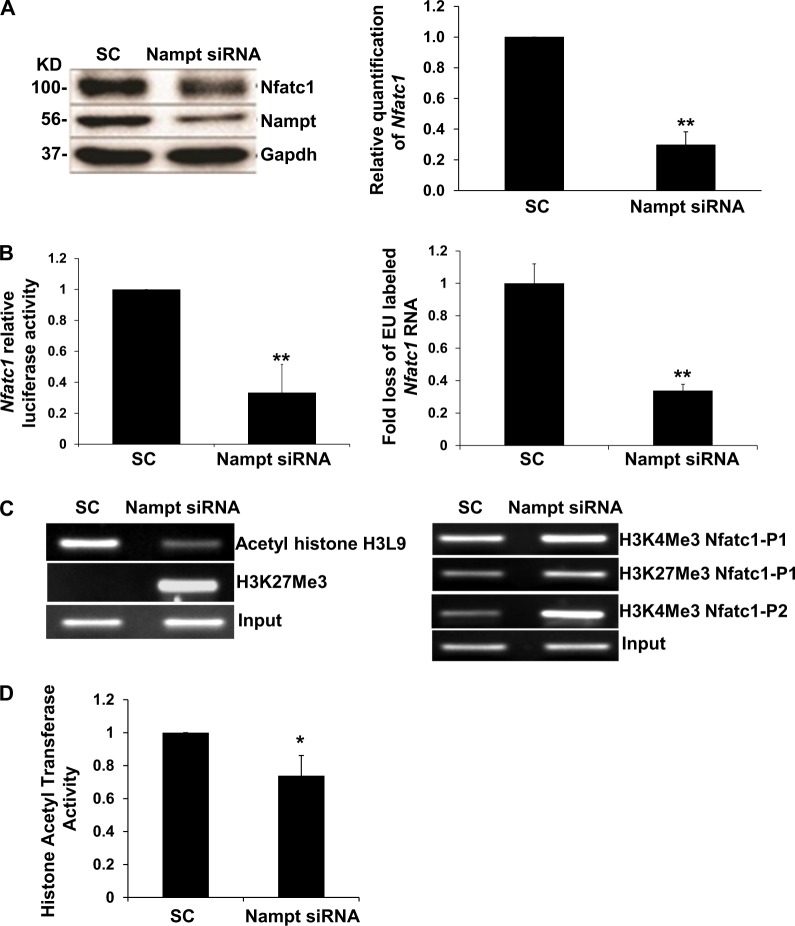
Nampt regulates the transcription of Nfatc1 by epigenetic remodeling of the promoter in RAW 264.7 cells. **a** Nfatc1 expression is inhibited by the *Nampt* siRNA treatment. Nfatc1 protein expression in Nampt knocked down RAW 264.7 cells was measured by western blot. Equal amounts (20 µg) of whole cell lysates were immunoblotted for Nfatc1, Nampt, and Gapdh. Relative quantification of *Nfatc*1 gene expression in scrambled control and *Nampt* siRNA transfected RAW 264.7 cells (*N* = 4). **b** RAW 264.7 cells were co-nucleofected with scrambled control or Nampt siRNA and an Nfatc1-Luc reporter construct. Relative Nfatc1 luciferase activities were normalized to non-stimulated scrambled siRNA transfected control cells. Each determination represents the average of three independent experiments. Relative quantification of *Nfatc*1 mRNA following nascent RNA capture in RAW 264.7 cells nucleofected with either SC siRNA or Nampt siRNA. Transcriptional rates presented as fold loss of EU labeled control RNA. **c** ChIP-PCR analysis of RAW 264.7 cells nucleofected with either scrambled siRNA or Nampt siRNA. The P1 region of the Nfatc1 promoter was amplified following immunoprecipitation with the acetyl histone lysine 9 antibody and P1 and P2 promoter regions were amplified following immunoprecipitation with H3K4Me3 and H3K27Me3 antibodies. **d** Histone acetyltransferase activity (HAT) in scrambled control and Nampt siRNA transfected RAW 264.7 cells. *P < 0.05; **P < 0.005

To investigate the mechanism by which Nampt-deficiency attenuated macrophage differentiation into TRAP^+^ cells, we examined the expression of Nfactc1, an essential transcriptional regulator of osteoclast differentiation^10^. Silencing of Nampt expression by siRNA in RAW 264.7 cells inhibited expression of Nfatc1 protein and mRNA (Fig. 3a). To determine the regulatory mechanism of Nfatc1 expression, we tested mRNA stability in response to decreased Nampt levels. Although there was a decrease in mRNA stability, it was not sufficient to account entirely for the loss in protein levels (data not shown). Therefore, we investigated *Nfatc*1 transcription using luciferase reporter and nascent RNA capture assays. The relative luciferase activity decreased significantly in RAW 264.7 cells co-transfected with the *Nfatc*1 promoter luciferase reporter and Nampt siRNA relative to the *Nfatc*1 promoter luciferase reporter and scrambled siRNA control cells (Fig. 3b). Nascent RNA capture in RAW 264.7 cells validated further that synthesis of *Nfatc*1 mRNA required Nampt expression (Fig. 3b).

Epigenetic re-modeling of the *Nfatc*1 promoter plays a critical role in Nfatc1 expression^11^. Therefore, we performed ChIP-PCR analyses in Nampt-deficient RAW 264.7 cells to characterize the transcriptional regulation of the *Nfatc*1 promoter. The interaction of acetylated histones, which represents open chromatin, with the *Nfatc*1 P1 promoter decreased following Nampt knockdown (Fig. 3c). Conversely, the presence of methylated histones, which represents closed chromatin, increased at the *Nfatc*1 P1 and P2 promoter regions following *Nampt* siRNA knockdown compared with scrambled siRNA transfected controls (Fig. 3c). These observations corresponded with decreased histone acetyltransferase (HAT) activity in RAW 264.7 cells subjected to Nampt knockdown (Fig. 3d). The epigenetic remodeling was consistent with the decreased transcriptional activity observed by luciferase reporter and nascent RNA capture assays (Fig. 3b).

**Fig. 4:**
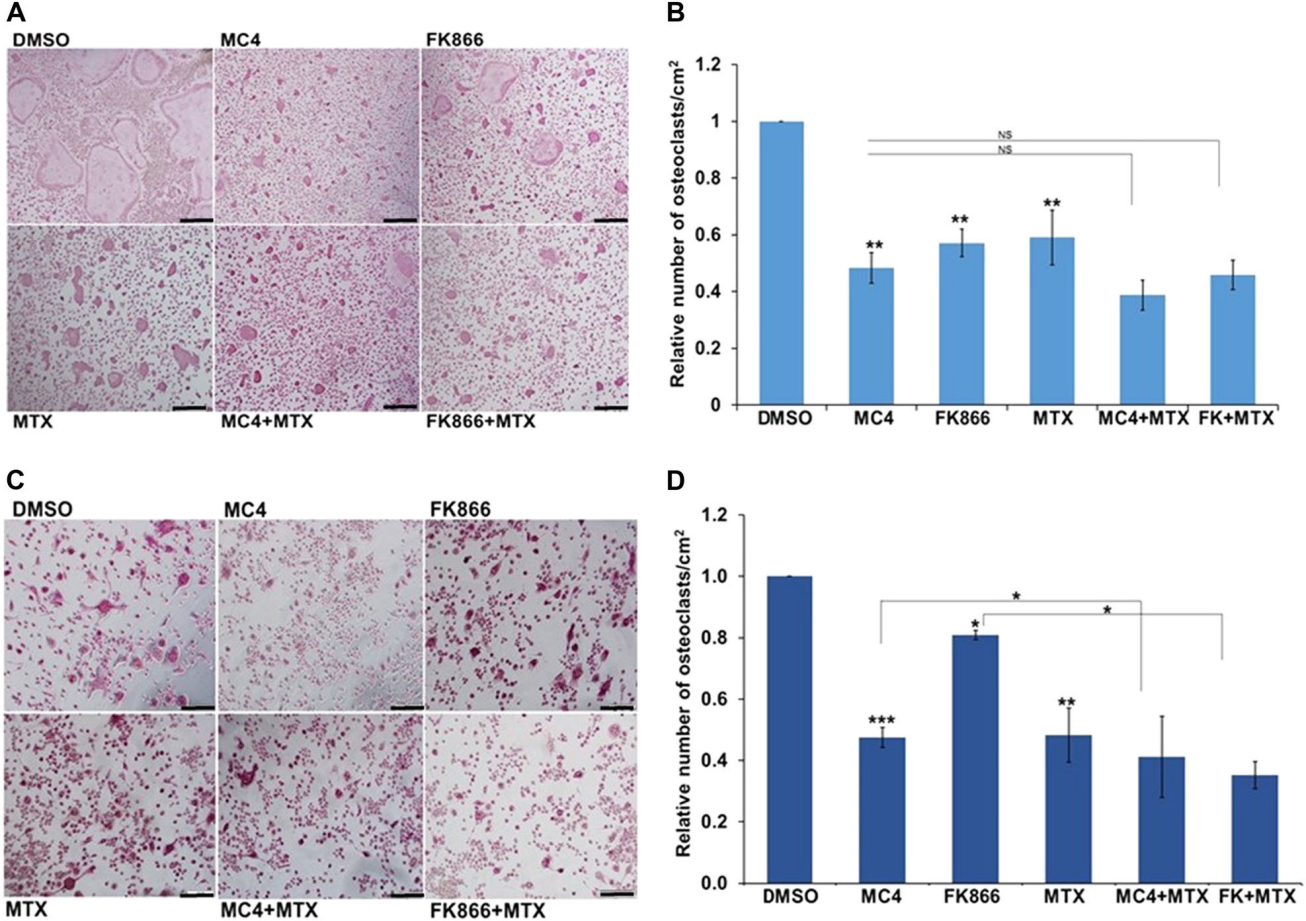
Inhibition of Rankl induced osteoclasts differentiation by Nampt enzyme inhibitors MC4, FK866 with or without MTX in RAW 264.7 and BMM. **a** RAW cells were pretreated with 2 nM MC4 or FK866 or 50 nM MTX for 3 h and then stimulated with Rankl (100 ng/ml) for 5 days. TRAP staining was performed using a TRACP and ALP double staining kit. **b** The frequency of TRAP-positive cells expressed as mean of triplicate determinations ± SD. **c** BMM were isolated from wild-type mice pretreated with 2 nM MC4 or FK866 or 50 nM MTX for 3 h and then stimulated with Rankl (100 ng/ml) for 5 days. TRAP staining was performed using a TRACP and ALP double staining kit. **d** The frequency of TRAP-positive cells expressed as mean of triplicate determinations ± SD. *P < 0.05; **P < 0.005; ***P < 0.001

To determine if Nampt enzymatic activity was required for the Nampt–NfatC1–osteoclastogenesis pathway, we treated RAW 264.7 and *Nampt*^+/+^ BMM with the enzymatic inhibitors, FK866^12^ and MC4^13^ during Rankl-induced differentiation. Formation of TRAP^+^ cells was decreased significantly in RAW 264.7 cells treated with FK866 and MC4 relative to the DMSO-vehicle control cells (Fig. 4a, b). Differentiation of *Nampt*^+/+^ BMM also decreased significantly in response to FK866 and MC4 relative to the control cells (Fig. 4c, d). In both RAW 264.7 cells and *Nampt*^+/+^ BMM, MC4 was as effective as MTX in blocking the formation of TRAP^+^ cells. The combination of MC4 and MTX in RAW 264.7 and *Nampt*^+/+^ BMM significantly blocked osteoclast differentiation compared with MC4 or MTX treatment alone (Fig. 4b, d). These findings strongly support the requirement of Nampt enzymatic activity to promote osteoclastogenesis.

### Transcriptomic profiling of whole ankle joints in CIA mice identifies genes and pathways associated with osteoclastogenesis

**Fig. 5:**
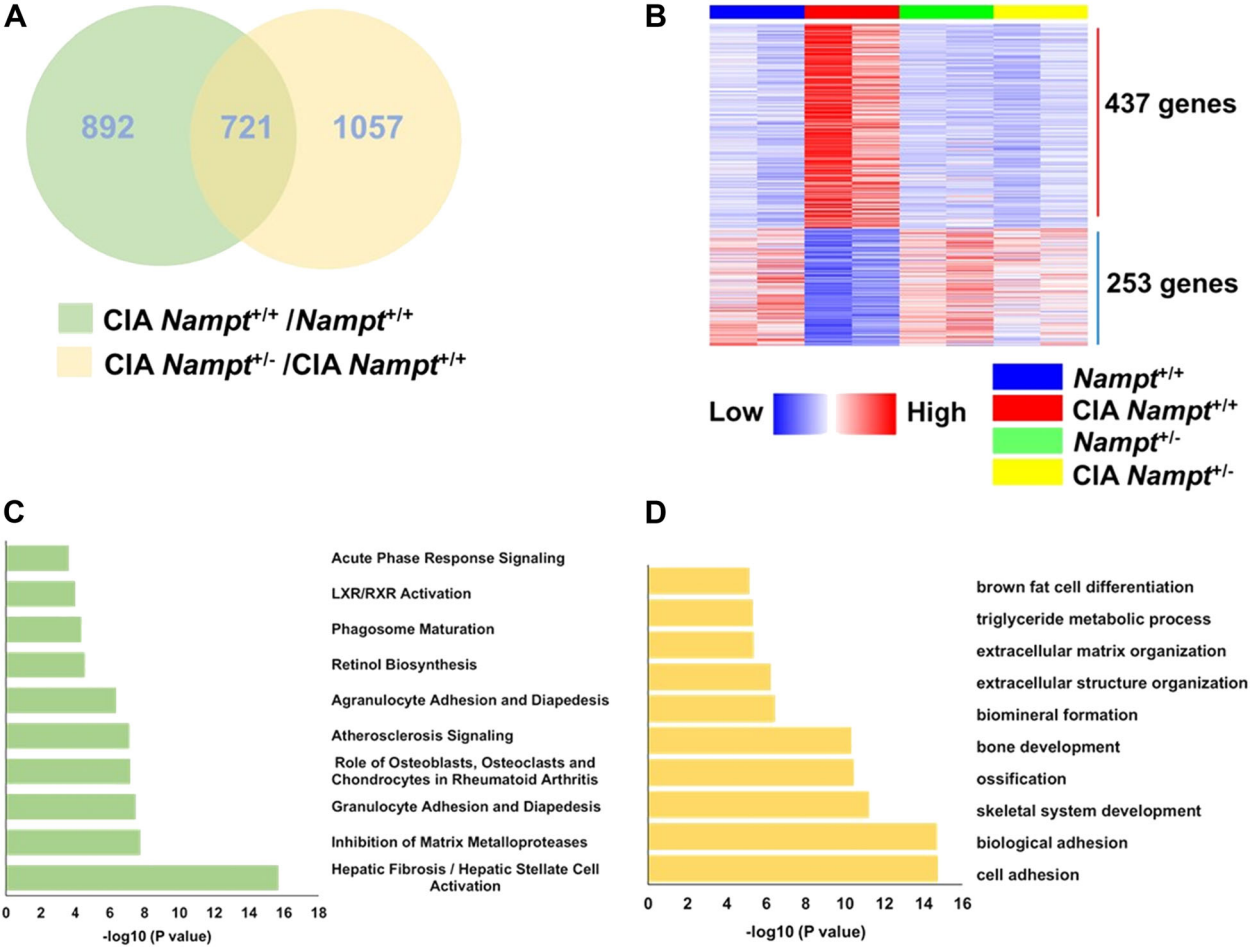
Transcriptional profiles of whole ankle joints of CIA in DBA/1J *Nampt*^+/+^ and *Nampt*^+/−^ mice. **a** Venn diagram representation of the 721 genes common to the 2 comparison groups. 690 genes were inversely regulated in CIA *Nampt*^+/+^/*Nampt*^+/+^ and CIA *Nampt*^+/−^/CIA *Nampt*^+/+^ mice (*P* < 0.05). **b** Heat map analysis of 690 significantly differentially expressed genes. Individual mouse samples are shown in columns and differentially expressed genes in rows. Red indicates an increased expression. Blue indicates a decreased expression. *N* = 2 for each group. **c** Top 10 canonical pathways as predicted by IPA for the 690 genes as described in Materials and methods. **d** The top 10 biological processes identified by the software program DAVID

To investigate the molecular mechanisms of Nampt in the pathogenesis of arthritis, we sequenced RNA isolated from whole ankle joints of *Nampt*^+/+^ and *Nampt*^+/−^ mice with and without CIA (Fig. 5). We initially determined the DEG in two comparison groups. There were 1613 DEG in CIA *Nampt*^+/+^ mice compared with non-CIA control *Nampt*^+/+^ mice and 1778 DEG in CIA Nampt^+/−^ mice compared with CIA *Nampt*^+/+^ mice. Comparison of these two lists identified 721 genes that were present in both groups (Fig. 5a), with 690 genes inversely regulated (Fig. 5b). We hypothesized these 690 genes were associated with Nampt’s role in the pathogenesis of CIA (Supplemental Table 1). The top five up-regulated protein-encoding genes in CIA *Nampt*^+/+^ ankle were *Oscar*, *Cxcl5*, *Tnn*, *Fam229a*, and *Atp6v0d2*, while the top five down-regulated genes were *Lep*, *Polr3g*, *Serpina3c*, *Pnpla3*, and *Nnat*. Although the majority of the 690 DEG in the CIA *Nampt*^+/+^ ankle were protein-coding genes, there were 10 non-coding RNA genes that were differentially expressed (Supplemental Table 1).

The finding that two osteoclast-specific genes, *Oscar* and *Atp6v0d2*, were among the top 5 up-regulated genes supported our observation that decreased Nampt expression limited osteoclastogenesis in both RAW 264.7 and BMM. Additionally, the osteoclast-specific genes *Mmp9*, *Acp5*, *Ctsk*, and *Dcstamp* were differentially expressed with increased expression in CIA *Nampt*^+/+^ mice (Supplemental Table 2). Calcium influx plays a critical role in the activation of Nfatc1^14^. We identified 19 significantly upregulated calcium metabolism genes within the 690 DEG (Supplemental Table 3). Our previous finding that knockdown of NAMPT expression significantly attenuated calcium influx into human pulmonary artery endothelial cells^15^, supports our hypothesis that decreased Nampt expression may inhibit osteoclast differentiation by inhibiting calcium influx via mediating expression of calcium metabolism genes.

To gain further insight into the biological functions associated with Nampt mediated pathogenesis of CIA, we submitted the 690 DEG for pathway analysis. These include several pathways associated with osteoclastogenesis, including the *Inhibition of Matrix Metalloproteases* and the *Role of Osteoblasts, Osteoclasts and Chondrocytes in Rheumatoid Arthritis* (Fig. 5c, Supplemental Table 4).

IPA analysis predicted target molecules in the dataset of 690 DEG that are either activated or inhibited by well-characterized upstream regulators. TNFα (5.575 activation *z*-score; 7.73E−46 *P*-value of overlap), TGFB1 (5.436; 9.4E−36), and LPS (5.985; 4.87E−32) are the top 3 activators, while the top 3 inhibitory regulators are the drugs dexamethasone (−3.074; 2.12E−34) and rosiglitazone (−4.791; 7.56E−24), and the kinase inhibitor PD98059 (−3.847; 1.33E−23) (Supplemental Table 5). Upstream regulator analysis also identified factors, such as cytokines (Tnfsf11, CSF1), transcriptional regulators (Fos, Nfatc1), and signaling proteins (Nfκb, Erk1/2, P38 Mapk, Src, P13k, Akt) that are known to promote osteoclast differentiation and bone resorption (Supplemental Table 6).

We next performed functional enrichment analyses for GO terms and KEGG to predict potential biological processes and pathways involved in the Nampt associated pathogenesis of CIA. The top biological processes linked with the 690 DEG include GO:0007155—cell adhesion, GO:0022610—biological adhesion, GO:0001501—skeletal system development, GO:0001503—ossification and GO:0060348—bone development (Fig. 5d, Supplemental Table 7). The top pathways identified by KEGG analysis include mmu04512:ECM—receptor interaction and mmu04142:Lysosome (Supplemental Table 8).

### Validation of RNA-seq result by RT-PCR assay of the selected targets as well as functional validation of GM26870, a long non-coding RNA

**Fig. 6:**
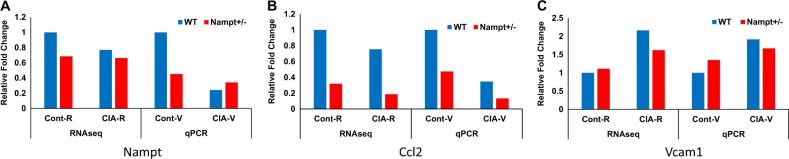
Validation of differential gene expression profiles determined by RNA seq via TaqMan™ qPCR analyses of selected targets. RNA isolation, RNA-seq, and RT-PCR were carried out as described in Materials and methods. All results of gene expression levels are expressed as relative fold changes. The relative fold changes represent mean values of technical replicates of *N* = 6 and biological replicates of *N* = 2. **a** Comparison of relative changes in *Nampt* expression levels for RNA-seq and qPCR. **b** Relative fold changes for *Ccl2*. **c** Relative fold changes *Vcam1*. All fold changes are relative to the wild-type (WT) control. The ∆∆CT was calculated by subtracting the mean ∆CT for the WT control group from the mean ∆CT for each experimental sample. *Gapdh* served as the endogenous control. Cont-R control RNA seq, CIA-R CIA RNA seq, Cont-V control TaqMan™ validation, CIA-V CIA TaqMan™ validation

To validate RNA-seq results, we performed RT-PCR assay of 3 selected targets: *Nampt*, Chemokine (C–C motif) ligand 12 (*Ccl12*), and Vascular cell adhesion protein 1 (*Vcam1*). In the control group, *Nampt* expression levels in *Nampt*^+/−^ mice were 40% of *Nampt*^+/+^ mice, while in the CIA group, *Nampt* expression levels were lower than those in the control group. The trend was similar to the RNA-seq result (Fig. 6a). *Ccl2* expression in *Nampt*^+/−^ mice was 40% of *Nampt*^+/+^ mice in the control group, while in the CIA group, *Ccl2* expression levels were lower than the control group with *Ccl2* expression in *Nampt*^+/−^ mice approximately 50% of *Nampt*^+/+^ mice. The trend was also similar to the RNA-seq result (Fig. 6b). Vcam1 expression in Nampt^+/−^ mice was about 20% higher than Nampt^+/+^ mice in the control group while in the CIA group, Vcam1 expression levels were all higher than in those in the control group. The trend was also similar to the RNA-seq result (Fig. 6c). Although we only assayed a limited number of targets, the RT-PCR analyses confirmed the RNA-seq results.

**Fig. 7:**
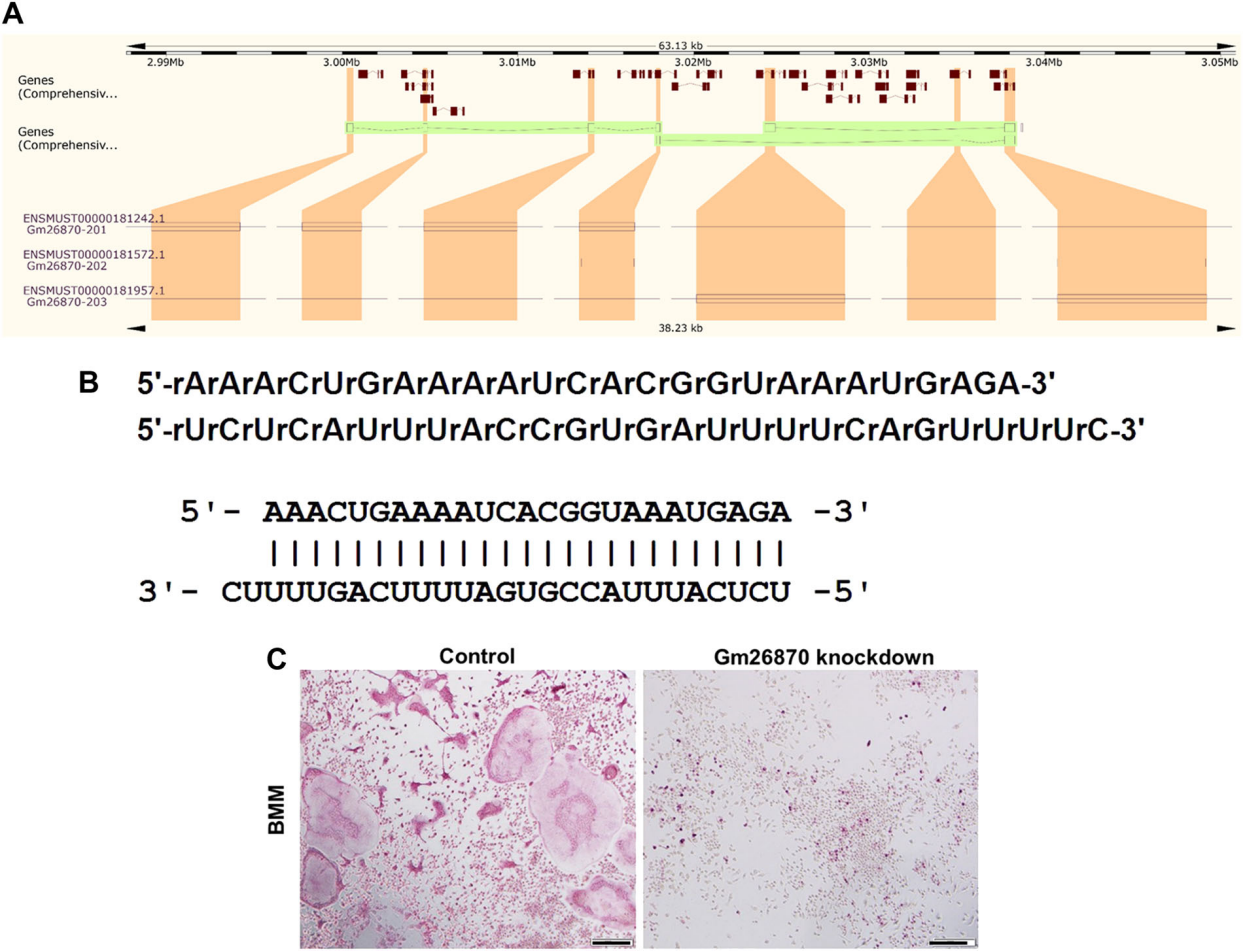
Knockdown of GM26870-201 expression blocks osteoblast formation from mouse bone marrow-derived macrophages. **a** Chromosome location of GM26870 and its encoding of three transcripts (splicing variants). GM26870 is located at Chromosome 9: 3,000,282–3,038,313 reverse strand. Shown in the left is the transcript GM26870-201 (1170 bp), shown in the middle is GM26870-202 (1170 bp), and shown in the right is GM26870-203 (1143 bp). Image is copied from the Ensembl website (http://useast.ensembl.org/Mus_musculus/Gene/Splice?db=core;g=ENSMUSG00000097312;r=9:3000282-3038313). **b** GM26870-201 dsiRNA sequence. The duplex dsiRNA consists of 25 bp in the forward strand and 27 bp in the reverse strand. **c** Effect of GM26870-201 knockdown on osteoclast formation in mouse BMM. Cell isolation and culture as well as Trap staining were carried out as described in Materials and methods. Shown clearly is that knockdown of GM26870-201 expression blocks osteoclast formation from mouse BMM

To validate the RNA-seq results functionally and to initiate signal transduction analyses of Nampt mediated pathways in CIA *Nampt*^+/−^ mice, we focused on GM26870, a differentially expressed long non-coding RNA (lncRNA), to examine whether it may be a component underlying the protective role of Nampt knockdown in arthritis. GM26870 (ENSMUSG00000097312), located on the reverse strand of Chromosome 9: 3,000,282–3,038,313, encodes 3 transcripts (splice variants) (Fig. 7a), which all have a length around 1100 bp. Among them, GM26879-201 is 1170 bp. We employed GM26870-201 dsiRNA synthesized by IDT (Fig. 7b) to knockdown GM26870-201 in mouse primary BMM to examine its effect on osteoclast formation. The knockdown of GM26870-201, successfully measured by RT-PCR (data not shown), blocked osteoclast formation (Fig. 7c) in mouse primary BMM.

### Overexpression of Nampt promotes osteoclast formation

**Fig. 8:**
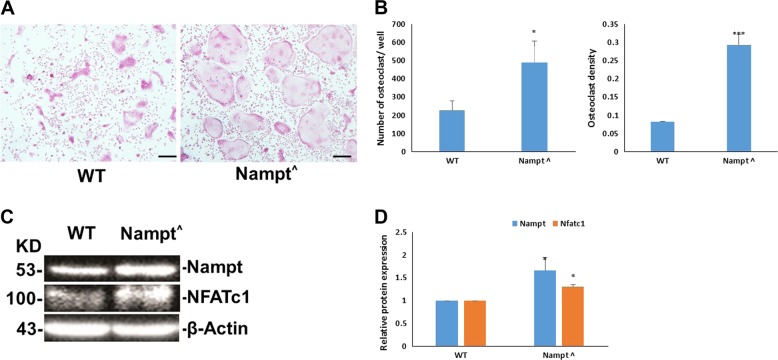
Overexpression of Nampt promotes osteoclast formation. Bone marrow cells were isolated from male DBA/1J *Nampt*^+/+^ and *Nampt*^OE^ mice and differentiated into osteoclasts. TRAP^+^ cells with >3 nuclei were counted as osteoclasts as described in Materials and methods. **a** Images of TRAP^+^ cells were captured under a light microscope (magnification, 10×). **b** Quantification of osteoclasts. **c** A representative image of western blot analysis of Nampt, Nfatc1, and β-Actin protein levels in *Nampt*^OE^ BMM. β-Actin was included as a loading control. **d** Quantification of Nampt and Nfatc1 protein levels. Data were presented as relative expression levels based on the value of *Nampt*^+/+^ wild type (WT) mice as 1. *P < 0.05 vs. *Nampt*^+/+^ mice and ***P < 0.001 vs. *Nampt*^+/+^ WT mice

Since knockdown of Nampt expression inhibits osteoclast formation, we hypothesized that overexpression of Nampt would promote osteoclast formation. To test this hypothesis, we isolated BMM from *Nampt*^+/+^ and *Nampt*^OE^ DBA/1J male mice and initiated cell differentiation into TRAP^+^ osteoclasts (Fig. 8a). As presented in Fig. 8b, the number of osteoclasts is as high as 450/well from *Nampt*^OE^ mice cells while only about 200/well in *Nampt*^+/+^ mice cells. Osteoclast density is above 0.25 in *Nampt*^OE^ mice cells vs. 0.1 in *Nampt*^+/+^ mice cells. Overexpression of Nampt in *Nampt*^OE^ mice cells was confirmed by western blotting (Fig. 8c). Nfatc1 was also upregulated in *Nampt*^OE^ mouse cells. Semi-quantitatively, Nampt and Nfatc1 expressions were 150% and 130% higher in *Nampt*^OE^ cells than those in *Nampt*^+/+^ cells, respectively. These results indicate that the overexpression of Nampt promotes osteoclast formation via upregulating Nfatc1.

## Discussion

In this study, we discovered that heterozygous knockdown of Nampt suppressed inflammatory bone destruction and disease progression in a CIA mouse model. We characterized one potential mechanism by which Nampt affects arthritis through its transcriptional regulation of the osteoclastogenesis essential transcription factor Nfatc1. Nampt expression was required for the epigenetic regulation of the Nfatc1 promoter and osteoclastogenesis. Finally, we performed transcriptome analysis of whole ankle joints isolated from *Nampt*^+/+^ and *Nampt*^+/−^ mice which demonstrated the enrichment of osteoclastogenesis genes and pathways and provided insight into the roles of Nampt in the pathogenesis of CIA. Selected targets were validated by RT-PCR for functional characterization. Our findings support our hypothesis that NAMPT is a genetic risk factor and potential therapeutic target for RA.

The role of Nfatc1 in osteoclastogenesis has been well characterized. However, the epigenetic regulation of Nfatc1 transcription by Nampt is a novel finding. Knockdown of Nampt expression inhibits Rankl expression (Fig. 2) while overexpression of Nampt upregulates Rankl expression (Fig. 7). Rankl is an upstream regulator of Nfatc1. RANKL belongs to the tumor necrosis factor superfamily and plays a critical role in osteoclast differentiation and bone destruction in RA^16^. Knockdown of Nampt expression inhibits Nfatc1 expression as well as Acp5, Dc-stamp, Cathepsin K mRNA expression which are known to be involved in osteoclastogenesis (Fig. 2). NFATc1 is required for sufficient osteoclast differentiation. It plays the role of a master transcription regulator of osteoclast differentiation^17^. The dysregulation of the NAMPT–RANKL–NFATC1–osteoclastogenesis axis may play a major role in bone erosion associated with chronic arthritis, which is underlying the role of NAMPT in the pathogenesis of RA. Two lines of evidence, luciferase reporter assays and RNA capture experiments, support that Nampt regulates the transcription of *Nfatc*1 (Fig. 3). Our ChIP-PCR analyses in Nampt-deficient RAW 264.7 cells found that the interaction of acetylated histones with the *Nfatc*1 P1 promoter was decreased (Fig. 3c) while that of methylated histones was increased at the *Nfatc*1 P1 and P2 promoter regions. These observations corresponded with decreased HAT activity in RAW 264.7 cells subjected to Nampt knockdown (Fig. 3d). Histone acetylation is correlated with gene expression activation^18^. The presence of methyl moieties inhibits gene expression^19^. Knockdown of Nampt expression inhibits histone acetylation while enhancing histone methylation of the Nfatc1 gene promoter and hence its expression. Our study provides the first evidence that Nampt knockdown inhibits osteoclast formation via epigenetic inhibition of Nfatc1 gene expression. The epigenetic remodeling was consistent with the decreased transcriptional activity observed by luciferase reporter and nascent RNA capture assays (Fig. 3b). Our finding that the formation of Rankl-stimulated TRAP^+^ cells was blocked by treatment with the small molecule Nampt inhibitors, FK866^12^ or MC4^13,20^, provides evidence for the requirement of NAD^+^ in osteoclastogenesis. In addition, the increased inhibitory effect of an MC4 + MTX combination relative to individual treatments of each drug, support our earlier work which demonstrated that the inhibition of NAMPT potentiated the effectiveness MTX^21^.

A few RNA-seq studies have been applied to rheumatic diseases^22–27^. In the present study, RNA-seq analysis of mouse whole ankle joints identified 690 genes that are associated with Nampt’s role in CIA pathogenesis. They revealed not only “usual suspects” in the pathogenesis such as upregulation of inflammatory activator (TNFα, TGFB1, and LPS), transcriptional regulators (Fos, Nfatc1), and signaling proteins (Nfκb, Erk1/2, P38 Mapk, Src, P13k, Akt) that are known to promote osteoclast differentiation and bone resorption but also provided a number of new targets and novel insight into the role of Nampt in the pathogenesis of arthritis. First, we found that two osteoclast-specific genes, *Oscar* and *Atp6v0d2*, were among the top 5 up-regulated genes in CIA *Nampt*^+/+^ mice vs. CIA *Nampt*^+/−^ mice. OSCAR–collagen interaction stimulates RANK-dependent osteoclastogenesis^28^. OSCAR can play a proinflammatory role in monocyte-derived cells and contribute crucially on multiple levels to RA pathogenesis. The RANK/c‑Fos/ATP6V0D2 signaling pathway is an important pathway in the osteoclastogenesis^29^. Second, functional enrichment analyses for GO terms and KEGG to predict potential biological processes and pathways involved in the Nampt associated pathogenesis of CIA has identified many new targets in biological processes: GO:0001501—skeletal system development, GO:0001503—ossification, and GO:0060348—bone development (Fig. 5d, Supplemental Table 7). Third, a number of non-coding RNAs was differentially expressed (Supplemental Table 7) and they are novel targets which may underlie Nampt’s role in the pathogenesis of RA. This short discussion just scratches the surface of our RNA-seq data. It should be mentioned that we validated RNA results by RT-PCR of selected targets: *Nampt*, *Ccl2*, and *Vcam1* (Fig. 6), support the validity of our RNA-seq results. Thus our RNA-seq data provide a rich resource for us and others to further experimentation to characterize new targets in the pathogenesis of RA in the future. We also functionally validated one of the differentially expressed lncRNA, GM26870, and found that knockdown of GM26870 inhibited osteoclast formation (Fig. 8). It may in part be among the Nampt mediated pathways. lncRNAs can function as modular scaffolds to specify higher-order organization in RNP complexes and in chromatin states^30^. It forms extensive networks of ribonucleoprotein (RNP) complexes with numerous chromatin regulators. It is increasingly recognized that lncRNAs play critical roles in multiple biological processes across all kingdoms of life^30^. GM26870’s biological role is thus far unknown. Our study here provides the first gleam into GM26870’s function or pathological role.

In conclusion, we demonstrated that decreased Nampt expression attenuates inflammatory bone loss in a *Nampt*^+/−^ CIA mouse model. In vitro assays revealed impaired osteoclastogenesis in Nampt-deficient RAW 264.7 and BMM which corresponded with epigenetic suppression of *Nfatc*1 transcription and may provide a potential mechanism by which the Nampt–NfatC1–osteoclastogenesis pathway promotes arthritis. RNA-seq analysis further supported these observations and uncovered new insights into the pathways associated with arthritis. In total, our findings suggest that NAMPT is a genetic risk factor and potential therapeutic target for RA.

## Materials and methods

### Antibodies and chemicals

RPMI 1640 and DMEM were purchased from Life Technologies. Lipopolysaccharide-*Escherichia coli* 055:B5 was obtained from Sigma Aldrich (#L6529; St. Louis, MO). TRACP and ALP double staining kit (#MK300) was purchased from Clontech (Mountain View, CA). Anti-TRAP1 antibody (#ab151239) was from Abcam (Cambridge, MA). Phospho-MAPK family antibody sampler kit (#9910), pNF-κB p105 (#4806), pNF-κBp65 (#3033), Acetyl-Histone H3 (Lys9) (#9649), Tri-Methyl-Histone H3 (Lys27) (#9733) antibodies, Simple ChIP Enzymatic Chromatin IP kit (#9003), and cell lysis buffer (#9803) were purchased from Cell Signaling Technology (Beverly, MA). Recombinant Mouse M-CSF (#576406) and purified anti-NFATc1 antibody (#649601) were purchased from Biolegend (San Diego, CA). GAPDH (#sc25778), anti-Mouse and anti-Rabbit secondary antibodies were from Santa Cruz Biotechnology (Santa Cruz, CA). Nampt siRNA (Stealth_116; 5′-CCACCCAACACAAGCAAAGUUUAUU-3′) and Scrambled control siRNA (stealth_con 116, 5′-CCACAACAACAAACGUUGAUCCAUU-3′), Click-iT Nascent RNA capture kit (#C10365), Superscript III first strand synthesis supermix for qRT-PCR (#11752-050), Superscript VILO cDNA synthesis Supermix (#11754–050), and mouse Rankl recombinant protein (#RP-8601) were from (ThermoFisher Scientific, Waltham, MA). TaqMan® gene expression assays for *Nfatc1* (Mm00479445_m1), *Acp5* (mCG22832), and TaqMan® gene expression master mix (#4369016) were purchased from Applied Biosystems (Foster City, CA). SF cell line 4D Nucleofector X kit was from Lonza (Alpharetta, GA). Anti-Nampt antibody (#A300–372A) was purchased from Bethyl Laboratories (Montgomery, TX).

#### Cell culture

The murine macrophage cell line RAW 264.7 (TIB- 71, ATCC®) was maintained in RPMI 1640 media containing 10% FBS and antibiotics. All cells were grown at 37 °C, 5% CO_2_.

### Animal studies

Mice were maintained in an Association for Assessment and Accreditation of Laboratory Animal Care accredited institution in a temperature-controlled, pathogen-free facility with a 12 h light/dark cycle. The mice were gang housed with free access to food (Irradiated Teklad Global 18% Rodent Diet, Envigo, cat# 2918.15) and water. DBA/1J mice (The Jackson Laboratory) were crossed with C57BL/6J *Nampt*^+/−^ mice^31^. Offspring were backcrossed to DBA/1J for >10 generations to develop congenic DBA/1J *Nampt*^+/−^. To induce arthritis, 10-week-old, male mice were immunized with bovine collagen type II (CII, 100 µg, Chondrex) in Complete Freund’s Adjuvant (CFA, 100 µg *Mycobacterium tuberculosis*, Chondrex) by intradermal injection at the base of the tail. A collagen booster (100 µg) in Incomplete Freund’s Adjuvant (IFA, Chondrex) was administered at day 21^8^. Mice were evaluated for the onset of inflammation and scored (scale 0–4 per paw) as described by Brand et al.^8^. Mice were scored twice per week for 10 weeks. At the end of the 10-week period, the animals were euthanized and tissue was isolated for analysis. Circulating serum levels of anti-mouse CII antibody (Chondrex) and Nampt (AdipoGen®) were determined by ELISA. The left hind limb was scanned with a vivaCT40 (Scanco) in the Skeletal Imaging and In vitro-In vivo Mechanical Core in the University of Missouri Kansas City, School of Dentistry, as described previously^32^. The right hind limb was collected for RNA isolation and gene expression analyses.

Overexpression (Nampt^OE^) mice were generated in our lab as described previously^33^.

### Isolation of bone marrow-derived macrophage

Isolation of BMM was performed as previously described^34^.

### In vitro osteoclastogenesis

Osteoclasts were generated from bone marrow as described previously^35^.

### siRNA mediated Nampt knockdown

RAW 264.7 cells were 4D-nucleofected with Nampt and scrambled siRNA (50 nM/2 × 10^6^ cells) in SF solution (Amaxa). Cells were seeded at 5 × 10^4^ nucleofected cells/well/24-well plate for TRAP staining and 1 × 10^5^ cells/well/6-well plate for protein isolation.

### Protein extraction and western blot analysis

Protein extraction and western blot analysis were carried according to our previous procedure^33^.

### ChIP assay

SimpleChIP® Enzymatic Chromatin IP assays (Cell Signaling Technology) with Acetyl-Histone H3 (Lys9) and Tri-Methyl-Histone H3 (Lys27) antibodies were utilized according to the manufacturer’s instruction. Immunoprecipitated DNA was reverse cross-linked, purified and analyzed by PCR (primers: NFATc1-617-F 5′-GGAAGCCTGCGATTTTACAT-3′, NFATc1-426-R 5′-ACGAAACGGGAAGGAAAG-3′).

### Histone acetyltransferase (HAT) assay

HAT enzyme activity was quantified in Nampt-deficient and scrambled control cells using an EpiQuik HAT assay (EpiGentek) according to the manufacturer’s instruction.

### Luciferase reporter assays

Luciferase reporter assays were performed as described previously^34^.

### RNA isolation, quantitative RT-PCR, and nascent RNA capture

RNA isolation, quantitative RT-PCR, and nascent RNA capture were performed as we previously described^36^.

### RNA-seq

RNA was isolated from flash frozen whole ankle joints collected after the 10-week CIA observation period. cDNA sequencing libraries were prepared with an Illumina TruSeq Stranded Total RNA Sample Prep Kit and subjected to 2 × 101 paired-end sequencing as described previously^3^. Mapping of RNA-seq reads and transcript assembly and abundance estimation were conducted using Tuxedo Suite pipeline (TopHat v1.3.0/Bowtie v0.12.7/Cufflinks v1.0.3) and reported in Fragments Per Kilobase of exon per Million fragments mapped (FPKM). To identify genes which were differentially expressed, fold changes for each gene were calculated by dividing the average FPKM for the case by the average FPKM for the control. We determined fold changes for two comparison groups: (1) CIA *Nampt*^+/+^ mice compared with non-CIA control *Nampt*^+/+^ mice, and (2) CIA *Nampt*^+/−^ mice compared with CIA *Nampt*^+/+^ mice.

### Functional pathway analysis

Kyoto Encyclopedia of Genes and Genomes (KEGG) analyses to identify cellular pathways and biological processes associated with differentially expressed genes (DEG) were performed with DAVID^37^. Ingenuity Pathway Analysis (IPA) (Ingenuity Systems) predicted functional and canonical pathways.

### Statistics

Statistical analyses were performed with Sigma Stat (v4.0, Systat Software, Inc.). Results were expressed as mean ± SD. *P* < 0.05 was considered statistically significant.

## Supplementary information

Epigenetic regulation of NfatC1 transcription and osteoclastogenesis by nicotinamide phosphoribosyl transferase in the pathogenesis of arthritis

## Data Availability

The RNA-seq data have been deposited to Gene Expression Omnibus (http://www.ncbi.nlm.nih.gov/geo; accession number GSE121793).

## References

[1] Scott DL, Wolfe F, Huizinga TW (2010). Rheumatoid arthritis. Lancet.

[2] Glant TT, Mikecz K, Rauch TA (2014). Epigenetics in the pathogenesis of rheumatoid arthritis. BMC Med..

[3] Zhang LQ (2017). Metabolic and molecular insights into an essential role of nicotinamide phosphoribosyltransferase. Cell Death Dis..

[4] Zhang LQ, Heruth DP, Ye SQ (2011). Nicotinamide phosphoribosyltransferase in human diseases. J. Bioanal. Biomed..

[5] Busso N (2008). Pharmacological inhibition of nicotinamide phosphoribosyltransferase/visfatin enzymatic activity identifies a new inflammatory pathway linked to NAD. PLoS One.

[6] Présumey J (2013). Nicotinamide phosphoribosyltransferase/visfatin expression by inflammatory monocytes mediates arthritis pathogenesis. Ann. Rheum. Dis..

[7] Seki N (1988). Type II collagen-induced murine arthritis. I. Induction and perpetuation of arthritis require synergy between humoral and cell-mediated immunity. J. Immunol..

[8] Brand DD, Latham KA, Rosloniec EF (2007). Collagen-induced arthritis. Nat. Protoc..

[9] Vu TH (1998). MMP-9/gelatinase B is a key regulator of growth plate angiogenesis and apoptosis of hypertrophic chondrocytes. Cell.

[10] Winslow MM (2006). Calcineurin/NFAT signaling in osteoblasts regulates bone mass. Dev. Cell.

[11] Pham LV, Tamayo AT, Li C, Bueso-Ramos C, Ford RJ (2010). An epigenetic chromatin remodeling role for NFATc1 in transcriptional regulation of growth and survival genes in diffuse large B-cell lymphomas. Blood.

[12] Hasmann M, Schemainda I (2003). FK866, a highly specific noncompetitive inhibitor of nicotinamide phosphoribosyltransferase, represents a novel mechanism for induction of tumor cell apoptosis. Cancer Res..

[13] Lee MW, Sevryugina YV, Khan A, Ye SQ (2012). Carboranes increase the potency of small molecule inhibitors of nicotinamide phosphoribosyltranferase. J. Med. Chem..

[14] Kajiya H (2012). Calcium signaling in osteoclast differentiation and bone resorption. Adv. Exp. Med. Biol..

[15] Ye SQ (2005). Pre-B-cell-colony-enhancing factor is critically involved in thrombin-induced lung endothelial cell barrier dysregulation. Microvasc. Res..

[16] Tanaka S, Tanaka Y, Ishiguro N, Yamanaka H, Takeuchi T (2018). RANKL: a therapeutic target for bone destruction in rheumatoid arthritis. Mod. Rheumatol..

[17] Kim JH, Kim N (2014). Regulation of NFATc1 in osteoclast differentiation. J. Bone Metab..

[18] Verdone L, Agricola E, Caserta M, Di Mauro E (2006). Histone acetylation in gene regulation. Brief. Funct. Genom. Proteom..

[19] Razin A, Cedar H (1991). DNA methylation and gene expression. Microbiol. Rev..

[20] Huang P (2017). MC-PPEA as a new and more potent inhibitor of CLP-induced sepsis and pulmonary inflammation than FK866. Drug Des. Devel. Ther..

[21] Funk RS (2016). Nicotinamide phosphoribosyltransferase attenuates methotrexate response in juvenile idiopathic arthritis and in vitro. Clin. Transl. Sci..

[22] Heruth DP, Gibson M, Grigoryev DN, Zhang LQ, Ye SQ (2012). RNA-seq analysis of synovial fibroblasts brings new insights into rheumatoid arthritis. Cell Biosci..

[23] Shi L (2014). The SLE transcriptome exhibits evidence of chronic endotoxin exposure and has widespread dysregulation of non-coding and coding RNAs. PLoS ONE.

[24] Tandon M, Gallo A, Jang SI, Illei GG, Alevizos I (2012). Deep sequencing of short RNAs reveals novel microRNAs in minor salivary glands of patients with Sjögren’s syndrome. Oral Dis..

[25] Stone RC (2013). RNA-Seq for enrichment and analysis of IRF5 transcript expression in SLE. PLoS ONE.

[26] Moncrieffe, H. et al. Transcriptional profiles of JIA patient blood with subsequent poor response to methotrexate. *Rheumatology*. 10.1093/rheumatology/kex206 (2017). 10.1093/rheumatology/kex206 PMC5850489 28582527

[27] Choi S (2017). Transcription factor NFAT5 promotes macrophage survival in rheumatoid arthritis. J. Clin. Invest..

[28] Schultz HS (2016). OSCAR-collagen signaling in monocytes plays a proinflammatory role and may contribute to the pathogenesis of rheumatoid arthritis. Eur. J. Immunol..

[29] Zhu X, Zeng Z, Qiu D, Chen J (2018). Vgamma9Vdelta2 T cells inhibit immature dendritic cell transdifferentiation into osteoclasts through downregulation of RANK, cFos and ATP6V0D2. Int. J. Mol. Med..

[30] Rinn JL, Chang HY (2012). Genome regulation by long noncoding RNAs. Annu. Rev. Biochem..

[31] Hong SB (2008). Essential role of pre-B-cell colony enhancing factor in ventilator-induced lung injury. Am. J. Respir. Crit. Care Med..

[32] Mukai T (2015). Loss of SH3 domain-binding protein 2 function suppresses bone destruction in tumor necrosis factor-driven and collagen-induced arthritis in mice. Arthritis Rheumatol..

[33] Zhang LQ (2018). Novel protective role of nicotinamide phosphoribosyltransferase in acetaminophen-induced acute liver injury in mice. Am. J. Pathol..

[34] Ling M (2017). Epigenetic regulation of Runx2 transcription and osteoblast differentiation by nicotinamide phosphoribosyltransferase. Cell Biosci..

[35] Takayanagi H (2002). Induction and activation of the transcription factor NFATc1 (NFAT2) integrate RANKL signaling in terminal differentiation of osteoclasts. Dev. Cell.

[36] Bi G (2018). Up-regulation of SFTPB expression and attenuation of acute lung injury by pulmonary epithelial cell-specific NAMPT knockdown. FASEB J..

[37] da Huang W, Sherman BT, Lempicki RA (2009). Systematic and integrative analysis of large gene lists using DAVID bioinformatics resources. Nat. Protoc..

